# Primary broad ligament adenocarcinoma

**DOI:** 10.4322/acr.2020.176

**Published:** 2020-09-02

**Authors:** Arun Elangovan, Chinna Babu Dracham, Minu Chandra B Muddabhaktuni, Ariba Zaidi

**Affiliations:** a Postgraduate Institute of Medical Education and Research (PGIMER), Department of Radiotherapy & Oncology. Chandigarh, India.; b All India Institute of Medical Sciences, Department of Radiation Oncology. Rishikesh, India.; c Postgraduate Institute of Medical Education and Research (PGIMER), Department of Pathology. Chandigarh, India.

**Keywords:** Broad ligament, Carcinoma, Endometrioid, Mullerian Ducts

## Abstract

Primary broad ligament carcinoma is a very rare occurrence with 28 reported cases worldwide, to date. The epidemiology, treatment strategy and prognosis are still uncertain because of the scarcity of cases. Currently, all broad ligament carcinomas are managed similar to epithelial ovarian cancer. We report the case of a 43-year-old female with a prolonged complaint of abdominal pain and intermittent urinary retention, requiring frequent catheterization. She was diagnosed with obstructive right hydroureteronephrosis. The abdominal Contrast Enhanced Computed Tomography (CECT) revealed a well-defined heterogeneous lesion of 2.1х3х3.2cm size in the right lateral and posterior wall of the cervix. An ultrasound (USG)-guided Fine Needle Aspiration Cytology (FNAC) of the mass was done and it was suspected to be malignant. The patient underwent total abdominal hysterectomy, right salpingo-oophorectomy, pelvic lymph-nodal sampling, and peritoneal washing. Histological examination depicted an endometrioid adenocarcinoma of the broad ligament. She received adjuvant chemotherapy, followed by hormonal therapy. It has been five years since her surgery, and she is now alive and disease free.

## Abbreviations

CECT: Contrast Enhanced Computed Tomography

FATWO: Female Adnexal Tumor of probable Wolffian Origin

FIGO-IC: International Federation of Gynecology and Obstetrics - stage IC

OPD: Out-patient Department

STIC: Serous Tubal Intraepithelial Carcinoma

USG: Ultrasonography

## CASE REPORT

A 43-year-old female sought medical attention due to her complaints of frequent urinary voiding dysfunction requiring intermittent catheterization, and lower abdominal pain during micturition for a period of two years. She was first diagnosed with right hydroureteronephrosis and treated with the placement of a ureteric stent, without any relief of the urinary symptoms. Her past surgical history was significant for the exeresis of an endometrioma of the right ovary, 18 years before.

Her general physical examination as well as her per-abdominal, and per-vaginal examinations were normal. An abdominal CECT showed the right moderate hydroureteronephrosis with a double J (DJ) stent in situ. The uterus was bulky and heterogeneous, with the presence of few hypodense lesions within the uterine wall. A well-defined heterogeneous oval lesion of 2.1х3х3.2cm in the right lateral and posterior wall of the cervix, along with few foci of calcifications were found. The bilateral adnexa were normal. She subsequently underwent trans-vaginal USG-guided FNAC of the uterine cervical mass that was suspected to be malignant. A Papanicolaou smear test from the cervix was normal.

With the working diagnosis of a complicated uterine leiomyoma having chances of suspicious malignant transformation, the patient underwent total abdominal hysterectomy (TAH), right salpingo-oophorectomy (Right SO), pelvic lymph-nodal sampling, peritoneal cytology and ureteric stent replacement. Intraoperatively, a tumor with size of 4x4 cm was identified in the right broad ligament, posterolateral to the cervix. There was no ascites. Both the ovaries as well as the fallopian tubes were grossly normal. No lesions consistent with endometriosis or disease dissemination were found.

**Figure 1:**
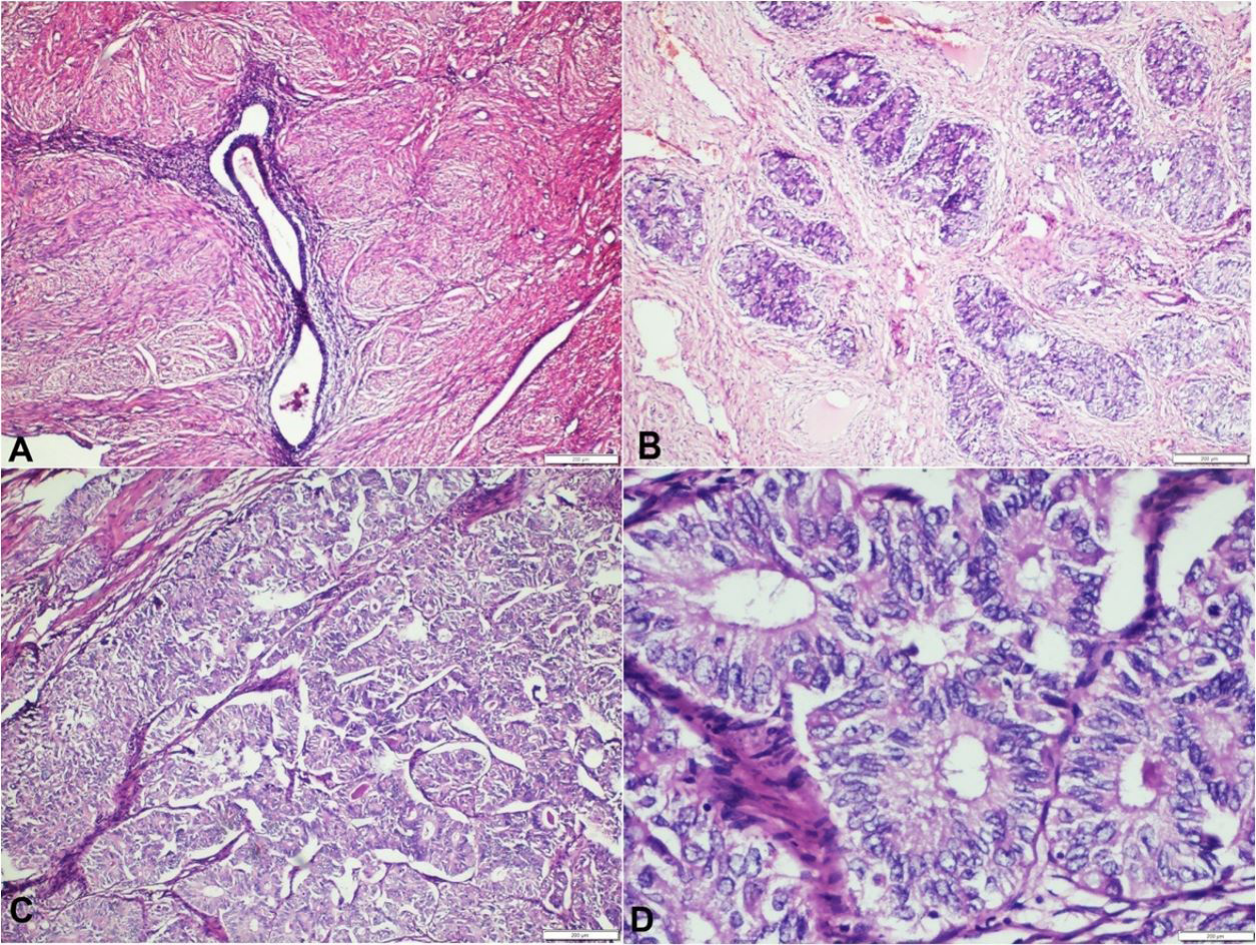
Photomicrograph of the tumor showing: **A** – Focus of endometrial glands and stroma within the adenomyoma in the broad ligament (H&E, 10X); **B** – Malignant transformation of glands into carcinoma (H&E, 10X); **C** – Tumor cells arranged in the form of glands, which are getting fused at places (H&E, 10X); **D** – Tumor cells which are moderately pleomorphic with fine chromatin, inconspicuous nucleoli and moderate amount of cytoplasm, Nuclear grade-1, Architectural grade-1 (H&E, 40X).

A gross examination of the surgical specimen showed a normal appearance of the uterus measuring 8х6х6 cm. However, a bulging mass of the right broad ligament measuring 2.5x1.5x2cm was attached to it. Post serial slicing, a single well-circumscribed, grey-white, firm tumor was identified in the broad ligament. The endometrial cavity, the endocervical and ectocervical mucosal surfaces, the right ovary and the fallopian tubes were all unremarkable. On microscopic examination the broad ligament mass showed adenomyoma with focal areas of malignant transformation. The tumor cells were arranged in the form of nests, glands and trabeculae, infiltrating the smooth muscle and reaching just beneath the ectocervix (Figure 1). Three internal iliac lymph nodes were sampled that were free of tumor.

**Figure 2:**
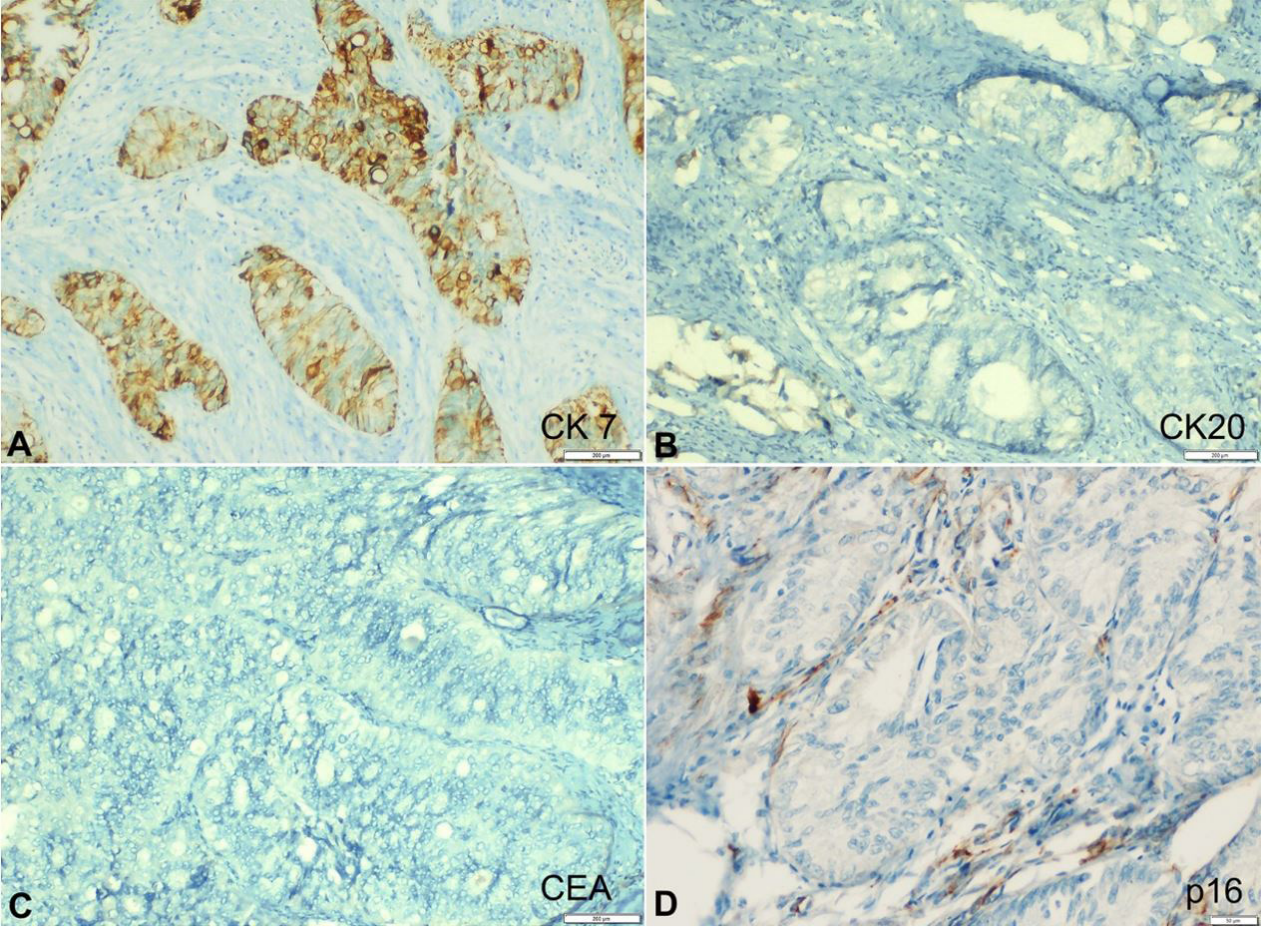
Photomicrograph of the tumor showing IHC profile: **A** – Strong membrane-cytoplasmic positivity for CK-7 (20X); **B** – Negative reaction for CK-20 (20X); **C** – Negative reaction for CEA (20X), D. Negative reaction for P16 (20X).

**Figure 3:**
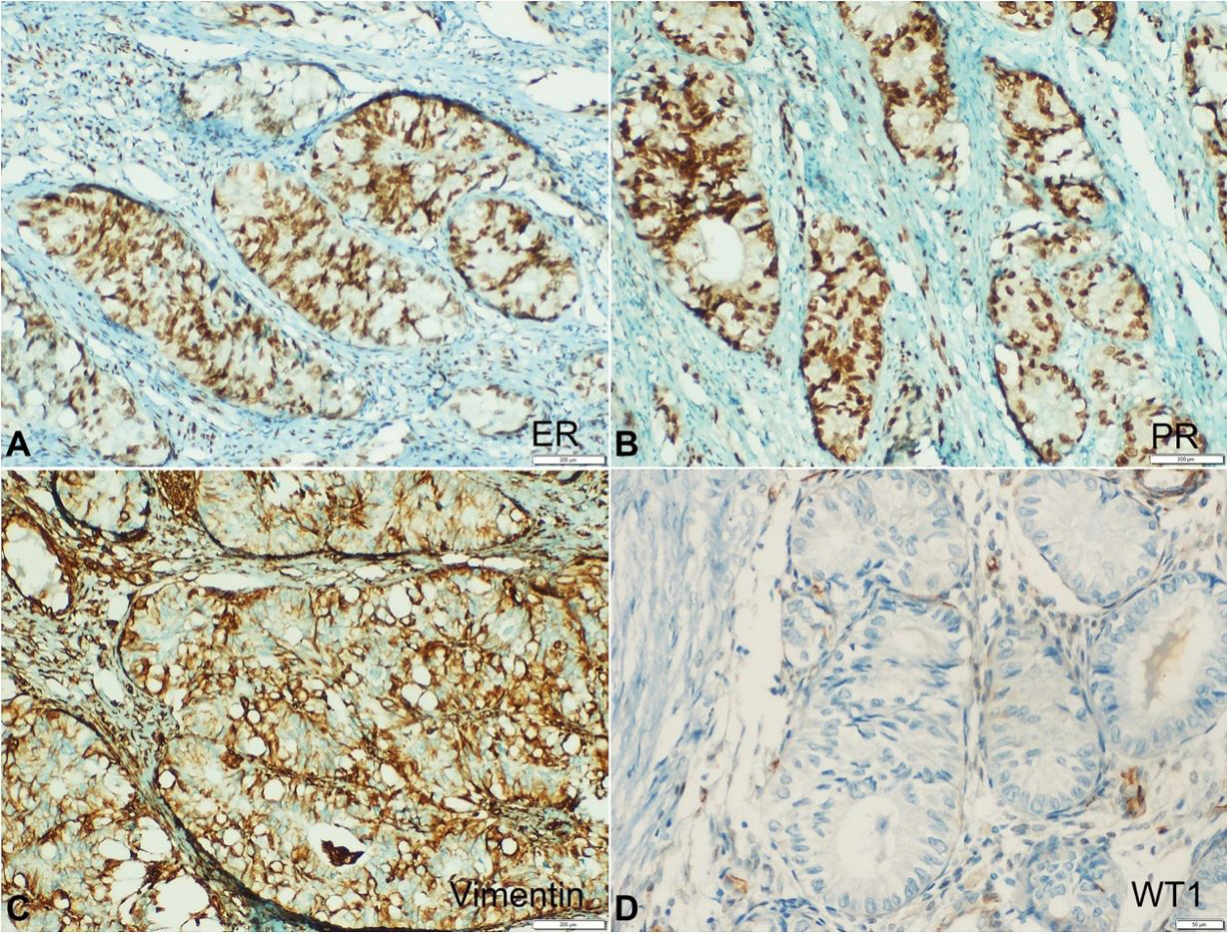
Photomicrograph of the tumor showing IHC profile: **A** – ER immunostain showing strong nuclear positivity for ER (20X); **B** – Strong nuclear positivity for PR immunostain (20X); **C** – Basolateral positivity for vimentin (20X); **D** – Negative reaction for WT1 (20X).

An immunohistochemistry (IHC) showed positivity for CK7 in the cytoplasm of the tumor cells, membranous positivity for vimentin and nuclear positivity for Estrogen (ER) and Progesterone Receptors (PR). IHC results for p16, CEA, CK20 and WT1 were negative (Figures 2 and 3).

The peritoneal washing done intraoperatively was suggestive of metastatic adenocarcinoma. The final histopathological diagnosis was endometrioid adenocarcinoma, grade I, arising from broad ligament adenomyoma, FIGO-IC.

The patient received adjuvant treatment with six cycles of paclitaxel and carboplatin chemotherapy, followed by hormonal therapy with tamoxifen 20mg daily. She completed five years of hormonal therapy during her latest visit, and is completely disease free clinically and radiologically. She is currently on a six-monthly clinical follow-up routine with yearly ultrasonography of the abdomen and the pelvis.

## DISCUSSION

We conducted a literature search in ‘PubMed’ and ‘Google Scholar’ using the keywords “broad ligament carcinoma and broad ligament endometrioid adenocarcinoma”. Additional papers were found through references in the primary articles (Table 1). In this section, we will review the collected literature to discuss origin, histopathological variants, role of IHC markers, clinical features and management approaches in the field of broad ligament carcinomas. Due to the scarcity of cases, no consensus therapy is established.

**Table 1:** Literature review on primary broad ligament carcinoma

Author	No. of cases	Histology	Tumor size (cm)	FIGO Stage	Surgery	Adjuvant treatment	Outcome
Miyoshi et al.^2^	2	Serous carcinoma Clear cell carcinoma	7.4×6.4×5.2	III	Modified Radical hysterectomy + BSO + PLND ^+^ Partial omentectomy	Chemotherapy	3 & 5 months – alive
Handa et al.^5^	1	Serous carcinoma	3.9 × 3.2	I	TVH+ BSO + PLND + PAND	Chemotherapy	36 months - alive
Brady et al.^6^	2	Papillary cystadenocarcinoma	-	-	TVH + BSO + PLND	-	-
Itani et al.^7^	1	Serous papillary adenocarcinoma	4.7x5.7	IC	TAH + BSO+ PLND + partial omentectomy + Peritoneal biopsy	Chemotherapy	18 months - alive
Aslani and Scully^8^	4	1 case: Clear cell 3 cases: Endometroid	10×6×6 11×8 (largest)	I (all)	TAH + BSO+ PLND+ partial omentectomy	Radiotherapy (one case) Chemotherapy (three cases)	6-18 months - alive
Schiller^9^	1	Clear cell carcinoma	-	I	TAH + BSO	Radiotherapy	24 months- alive
Teilum^10^	2	Clear cell carcinoma	10×8×8 (largest)	I & II	Enucleation	-	-
Thomason et al.^11^	1	Transitional cell carcinoma	6×4	I	TAH + BSO+ PLND + partial omentectomy + Peritoneal biopsy	Chemotherapy	20 months - alive
Mrad et al.^12^	1	Mucinous adenocarcinoma	7×3×3	I	TAH + Right SO + PLND + Partial omentectomy + Peritoneal biopsy + appendectomy	Radiotherapy given after recurrence	6 months
Hemalatha et al.^13^	1	Serous papillary carcinoma with foci of transitional differentiation	-	II	TAH + BSO+ PLND	Chemotherapy	15 months - alive
Torres et al.^14^	1	Clear cell adenocarcinoma	2.8 × 2.9 × 2.8	II	TAH + BSO+ PLND + partial omentectomy + Peritoneal biopsy	Chemotherapy	3 months - alive
Nagar et al.^15^	1	Papillary serous cystadenocarcinoma	7 × 7 × 6	III	TAH + BSO+ PLND	Chemotherapy	15 months - alive
Kaur et al.^16^	1	Endometroid adenocarcinoma	13 × 8 × 5	IIIC	TAH + BSO+ PLND + partial omentectomy + Peritoneal biopsy	Chemotherapy	3 months - alive
Our case	1	Endometroid adenocarcinoma	2.5×1.5×2	IC	TAH + Left SO + PLND + Peritoneal biopsy	Chemotherapy + Hormonal therapy	5 years – alive

BSO: Bilateral salpingo oophorectomy, PAND: Para-aortic lymph node dissection, PLND: Pelvic lymph node dissection, SO: Salpingo-oophorectomy, TAH: Total abdominal hysterectomy, TVH: Total vaginal hysterectomy.

Among all types of neoplasms arising in the broad ligament, leiomyoma and its variants are most common. Most of these lesions, either benign or malignant, clinically present with vague symptoms and are often encountered during a routine gynecological examination or an abdominal surgery for other reasons.

A primary broad ligament tumor was defined by Gardner et al.^1^ as a tumor with its primary location within or on the surface of the broad ligament, but entirely separated from the ipsilateral ovary, uterus and fallopian tube. Our case followed Gardner’s diagnostic criteria.

Most of the epithelial malignancies of the broad ligament are derived from the Mullerian remnants, including (i) serous, (ii) mucinous, (iii) clear cell, (iv) endometrioid, (v) borderline tumors and (vi) endometrial stromal sarcomas. Among all the reported carcinomas, serous adenocarcinoma, clear cell adenocarcinoma and endometrioid adenocarcinoma are the most common histologic variants^.^ ^2^ Other rare malignancies reported to arise from the broad ligaments are (i) Sex cord-stromal tumors, (ii) Neuroendocrine carcinomas (small cell and large cell), (iii) Sarcomas and (iv) Female Adnexal Tumor of probable Wolffian Origin (FATWO).^3^

The precise origin of broad ligament tumors is not known, but there are several hypotheses concerning its histogenesis. It is suggested that the broad ligament serous carcinomas arise from Serous Tubal Intraepithelial Carcinoma (STIC) lesions, similar to the ovarian high-grade serous carcinomas.^4^ Another hypothesis, like the ovarian carcinoma origin, explains the transformation of borderline and benign serous tumors into carcinoma. These benign serous tumors originate from inclusion cysts derived from the coelomic epithelium, which covers the ovarian and broad ligament surfaces. The primary endometrioid and clear cell adenocarcinomas of the broad ligament have their origin in the Mullerian ducts and arise from background endometriosis.^4^ In this case, there were no benign/borderline tumors or STIC-lesions. Although the patient was operated for right ovarian endometriosis 18 years prior, there was no endometriosis in the resected specimen during her current treatment. Most likely, the endometrioid carcinoma originated from an occult focus of endometriosis. The association of broad ligament carcinomas with endometriosis, as well as endosalpingiosis, was also reported in a few cases.^4^ ^,^ ^5^ The Mullerian-origin broad ligament tumors occur predominantly in reproductive women, but in the infertile and nulliparous, pointing out a possible hormonal role in the histogenesis of these tumors.^17^ In our case, the tumor expressed ER and PR receptors, which explain the connection between estrogenic stimulation and broad ligament tumors.

The Mullerian-origin broad ligament tumors and ovarian cancers can be confirmed by IHC. WT1, the most specific marker, is positive in 90% of the cases. IHC for CA-125 has a low specificity of 35%. The Mullerian-origin tumors also show positivity for ER, whereas the Wolffian-origin tumors show positivity for CD10, vimentin and calretinin, and negativity for ER. An IHC analysis by Brady et al.^6^ confirmed the above findings, but could not specifically suggest a Mullerian or Wolffian origin for the broad ligament tumors, as there occurs an overlap in the pattern of these markers.

As the broad ligament tumors are encased within the two layers of ligament, they tend to be asymptomatic during the early stages. Similarly, their rupture, progression and metastasis are delayed because of the lack of their own vascular supply, in contrast to the ovarian tumors.^5^ The main presenting features of the broad ligament tumors are vague abdominal pain and distension. Most symptoms depend on the tumor location. Our patient presented urinary complaints, which were thought to be a result of urinary bladder calculus. On further evaluation, she was found to have hydroureteronephrosis due to compression by the tumor on the ureter. The non-specific symptoms caused a delay in the diagnosis. The radiologic workup was an important diagnostic tool and guided the FNAC, which diagnosed the malignancy. Differing from the other reported cases,^7^ serum CA-125 was normal in this case.

There is a dearth of clinical experience in the management and prognosis of these tumors. Since the broad ligament is anatomically close to the ovary and the histological features of these two types of tumors are similar, their staging and management follow the epithelial ovarian cancer approach.

While up to 25% of the ovarian carcinomas are bilateral, the borderline and malignant lesions of the broad ligament are unilateral, and similar tumors do not accompany them in the ipsilateral ovary. Therefore, in a young woman who opts for preservation of ovarian function and in cases where the reproductive function is desired, fertility-preserving surgeries are justified.^8^ According to Arango et al.,^18^ a pelvic lymph-nodal dissection is recommended in the case of broad ligament carcinomas.

The exact role of the postsurgical treatment is not a consensus. Surgery alone,^6^ ^,^ ^10^ surgery followed by chemotherapy^5^ ^,^ ^7^ ^,^ ^11^ ^,^ ^13^ ^-^ ^16^ and/or radiotherapy^8^ ^,^ ^9^ ^,^ ^12^ have been used for treating primary broad ligament carcinoma. However, most authors advice adjuvant platinum-based chemotherapy (paclitaxel and carboplatin).^2^ ^,^ ^14^ ^-^ ^16^ In the present case, as the peritoneal cytology was positive for malignancy, the patient was planned for carboplatin and paclitaxel chemotherapy. This was followed by hormonal therapy because of the nuclear positivity for ER and PR. Prognosis of the broad ligament malignancies is also underestimated due to the very few cases. However, the cases diagnosed in an early stage tend to have a favorable outcome. Better and early recognition accompanied by molecular analysis and alternative treatment studies are needed to establish a fruitful management strategy.
